# 2-Chloro­methyl-1-methyl-1,3-benzimidazole

**DOI:** 10.1107/S1600536811028376

**Published:** 2011-07-23

**Authors:** Jie Han, Jun Zhang, Qi Yang, Ming-gao Zhao, Guang Fan

**Affiliations:** a Department of Pharmacology, School of Pharmacy, Fourth Military Medical University, Chang-le West Road 17, Xi’an 710032, Shaanxi, People’s Republic of China; b College of Chemistry & Chemical Engineering, Xianyang Normal University, Xianyang 712000, Shaanxi, People’s Republic of China

## Abstract

The title compound, C_9_H_9_ClN_2_, was prepared from the reaction of *N*-methyl­benzene-1,2-diamine and 2-chloro­acetic acid in boiling 6 *M* hydro­chloric acid. The  benzimidazole unit is approximately planar, the largest deviation from the mean plane being 0.008 (1) Å. The Cl atom is displaced by 1.667 (2) Å from this plane. The methyl group is statistically disordered with equal occupancy.

## Related literature

For the biological activity of benzimidazoles, see: Refaat (2010 ▶); Laryea *et al.* (2010 ▶); Horton *et al.* (2003 ▶); Ries *et al.* (2003 ▶); Spasov *et al.* (1999 ▶); Matsui *et al.* (1994 ▶); Porcari *et al.* (1998 ▶); Rath *et al.* (1997 ▶); Migawa *et al.* (1998 ▶). For a description of the Cambridge Structural Database, see: Allen (2002 ▶).
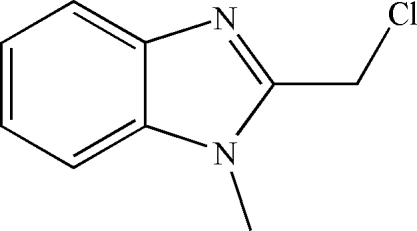


## Experimental

### 

#### Crystal data

C_9_H_9_ClN_2_
*M* *_r_* = 180.63
Triclinic, 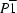

*a* = 6.607 (2) Å
*b* = 8.168 (2) Å
*c* = 8.925 (3) Å
α = 84.566 (3)°
β = 79.682 (4)°
γ = 68.134 (4)°
*V* = 439.6 (2) Å^3^
*Z* = 2
Mo *K*α radiation
μ = 0.38 mm^−1^
*T* = 296 K
0.37 × 0.29 × 0.18 mm

#### Data collection

Bruker SMART APEX CCD diffractometer
Absorption correction: multi-scan (*SADABS*; Bruker, 2002 ▶) *T*_min_ = 0.874, *T*_max_ = 0.937
2191 measured reflections
1523 independent reflections
1361 reflections with *I* > 2σ(*I*)
*R*_int_ = 0.018

#### Refinement

*R*[*F* ^2^ > 2σ(*F* ^2^)] = 0.042
*wR*(*F* ^2^) = 0.122
*S* = 1.06
1523 reflections
109 parameters
H-atom parameters constrained
Δρ_max_ = 0.20 e Å^−3^
Δρ_min_ = −0.32 e Å^−3^

### 

Data collection: *SMART* (Bruker, 2002 ▶); cell refinement: *SAINT* (Bruker, 2002 ▶); data reduction: *SAINT*; program(s) used to solve structure: *SHELXS97* (Sheldrick, 2008 ▶); program(s) used to refine structure: *SHELXL97* (Sheldrick, 2008 ▶); molecular graphics: *ORTEPIII* (Burnett & Johnson, 1996 ▶) and *ORTEP-3 for Windows* (Farrugia, 1997 ▶); software used to prepare material for publication: *SHELXL97*.

## Supplementary Material

Crystal structure: contains datablock(s) I, global. DOI: 10.1107/S1600536811028376/dn2705sup1.cif

Structure factors: contains datablock(s) I. DOI: 10.1107/S1600536811028376/dn2705Isup2.hkl

Supplementary material file. DOI: 10.1107/S1600536811028376/dn2705Isup3.cml

Additional supplementary materials:  crystallographic information; 3D view; checkCIF report

## Figures and Tables

**Fig. 1.**
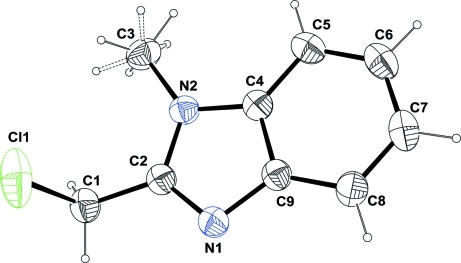
The asymmetric unit of (1) with the atom-labelling scheme. Displacement ellipsoids are drawn at the 30% probability level. H atoms are represented as small spheres of arbitrary radii.

## supplementary crystallographic information

### Comment

Benzimidazole and its derivatives are present in various bioactive compounds possessing antiparasitic, antimicrobial, and antifungal properties (Refaat, 2010; Laryea *et al.*, 2010; Horton *et al.*, 2003; Ries *et al.*, 2003; Spasov *et al.*, 1999; Matsui *et al.* 1994). They also play very important role in the synthesis of many natural products and synthetic drugs. Compounds possessing the benzimidazole moiety express significant activity against several viruses such as HIV (Porcari, *et al.*, 1998; Rath, *et al.*, 1997), Herpes (HSV-1) (Migawa, *et al.*, 1998), human cytomegalovirus (HCMV) and influenza. As a part of our ongoing investigations of benzimidazole derivatives, the title compound was synthesized and its crystal structure is reported herein.

The two fused rings forming the benzimidazole moiety are planar with the largest deviation from the mean plane being 0.008 (1)Å. The Cl atom is out of this plane by -1.667 (2)Å (Fig. 1). The methyl group is statistically disordered. The distances and angles within the methyl-benzimidazole agree with the values reported in the literature (43 hits found in the Cambridge Structural Database, Conquest, version 1.13; Allen, 2002).

The packing is only stabilized by electrostatic and van der Waals interactions.

### Experimental

For the preparation of the title compound *N*-methylbenzene-1,2-diamine (5.0 mmol) and 2-chloroacetic acid(6.0 mmol) was dissolved in 6 N hydrochloric acid (30.0 ml) and refluxed for 6 h. The reaction mixture was cooled in to room temperature, then neutralized with aqueous sodium hydroxide. The precipitate was filtered off and washed with cold water. The crude product was crystallized from ethanol to give white block-like crystals of the title compound.

### Refinement

All H atoms were fixed geometrically and treated as riding with C—H = 0.96 Å (methyl), 0.97 Å (methylene) and 0.93 Å (aromatic) with U_iso_(H) = 1.2U_eq_(C, aromatic or methylene) and U_iso_(H) = 1.5U_eq_(C, methyl).

### Crystal data

**Table:**

C_9_H_9_ClN_2_	*Z* = 2
*M**_r_* = 180.63	*F*(000) = 188
Triclinic, *P*1	*D*_x_ = 1.365 Mg m^−^^3^
Hall symbol: -P 1	Mo *K*α radiation, λ = 0.71073 Å
*a* = 6.607 (2) Å	Cell parameters from 2191 reflections
*b* = 8.168 (2) Å	θ = 2.3–25.1°
*c* = 8.925 (3) Å	µ = 0.38 mm^−^^1^
α = 84.566 (3)°	*T* = 296 K
β = 79.682 (4)°	Block, white
γ = 68.134 (4)°	0.37 × 0.29 × 0.18 mm
*V* = 439.6 (2) Å^3^	

### Data collection

**Table:**

Bruker SMART APEX CCD diffractometer	1523 independent reflections
Radiation source: fine-focus sealed tube	1361 reflections with *I* > 2σ(*I*)
graphite	*R*_int_ = 0.018
φ and ω scans	θ_max_ = 25.1°, θ_min_ = 2.3°
Absorption correction: multi-scan (*SADABS*; Bruker, 2002)	*h* = −7→5
*T*_min_ = 0.874, *T*_max_ = 0.937	*k* = −9→9
2191 measured reflections	*l* = −10→10

### Refinement

**Table:**

Refinement on *F*^2^	Primary atom site location: structure-invariant direct methods
Least-squares matrix: full	Secondary atom site location: difference Fourier map
*R*[*F*^2^ > 2σ(*F*^2^)] = 0.042	Hydrogen site location: inferred from neighbouring sites
*wR*(*F*^2^) = 0.122	H-atom parameters constrained
*S* = 1.06	*w* = 1/[σ^2^(*F*_o_^2^) + (0.0552*P*)^2^ + 0.1656*P*] where *P* = (*F*_o_^2^ + 2*F*_c_^2^)/3
1523 reflections	(Δ/σ)_max_ < 0.001
109 parameters	Δρ_max_ = 0.20 e Å^−^^3^
0 restraints	Δρ_min_ = −0.32 e Å^−^^3^

### Special details

**Table:**

Geometry. All esds (except the esd in the dihedral angle between two l.s. planes) are estimated using the full covariance matrix. The cell esds are taken into account individually in the estimation of esds in distances, angles and torsion angles; correlations between esds in cell parameters are only used when they are defined by crystal symmetry. An approximate (isotropic) treatment of cell esds is used for estimating esds involving l.s. planes.
Refinement. Refinement of *F*^2^ against ALL reflections. The weighted *R*-factor *wR* and goodness of fit *S* are based on *F*^2^, conventional *R*-factors *R* are based on *F*, with *F* set to zero for negative *F*^2^. The threshold expression of *F*^2^ > σ(*F*^2^) is used only for calculating *R*-factors(gt) *etc*. and is not relevant to the choice of reflections for refinement. *R*-factors based on *F*^2^ are statistically about twice as large as those based on *F*, and *R*- factors based on ALL data will be even larger.

### Fractional atomic coordinates and isotropic or equivalent isotropic displacement parameters (Å^2^)

**Table:**

	*x*	*y*	*z*	*U*_iso_*/*U*_eq_	Occ. (<1)
C1	0.2259 (4)	0.4372 (3)	0.6995 (2)	0.0610 (6)	
H1A	0.3424	0.4781	0.7109	0.073*	
H1B	0.0985	0.5393	0.6790	0.073*	
C2	0.1665 (3)	0.3458 (2)	0.8432 (2)	0.0498 (5)	
C3	−0.2214 (4)	0.3954 (3)	0.8004 (3)	0.0666 (6)	
H3A	−0.3405	0.3638	0.8563	0.100*	0.50
H3B	−0.1788	0.3444	0.7017	0.100*	0.50
H3C	−0.2683	0.5215	0.7888	0.100*	0.50
H3D	−0.1846	0.4560	0.7082	0.100*	0.50
H3E	−0.3463	0.4754	0.8628	0.100*	0.50
H3F	−0.2568	0.2983	0.7757	0.100*	0.50
C4	−0.0294 (3)	0.2393 (2)	1.0212 (2)	0.0488 (5)	
C5	−0.1859 (4)	0.1843 (3)	1.1144 (3)	0.0600 (6)	
H5	−0.3242	0.2069	1.0885	0.072*	
C6	−0.1250 (4)	0.0947 (3)	1.2471 (3)	0.0675 (6)	
H6	−0.2258	0.0574	1.3137	0.081*	
C7	0.0842 (4)	0.0581 (3)	1.2848 (3)	0.0673 (6)	
H7	0.1198	−0.0041	1.3751	0.081*	
C8	0.2384 (4)	0.1119 (3)	1.1915 (2)	0.0605 (6)	
H8	0.3773	0.0871	1.2173	0.073*	
C9	0.1803 (3)	0.2046 (2)	1.0572 (2)	0.0495 (5)	
Cl1	0.31643 (17)	0.29247 (10)	0.54306 (8)	0.1088 (4)	
N1	0.3008 (3)	0.2749 (2)	0.94299 (19)	0.0535 (4)	
N2	−0.0342 (3)	0.3294 (2)	0.88264 (18)	0.0498 (4)	

### Atomic displacement parameters (Å^2^)

**Table:**

	*U*^11^	*U*^22^	*U*^33^	*U*^12^	*U*^13^	*U*^23^
C1	0.0686 (13)	0.0587 (12)	0.0545 (12)	−0.0238 (11)	−0.0075 (10)	0.0029 (10)
C2	0.0509 (11)	0.0472 (10)	0.0489 (11)	−0.0161 (8)	−0.0037 (8)	−0.0048 (8)
C3	0.0599 (13)	0.0680 (14)	0.0736 (15)	−0.0195 (11)	−0.0252 (11)	0.0030 (11)
C4	0.0505 (10)	0.0436 (10)	0.0495 (11)	−0.0146 (8)	−0.0031 (8)	−0.0077 (8)
C5	0.0550 (12)	0.0568 (12)	0.0676 (14)	−0.0235 (10)	0.0018 (10)	−0.0068 (10)
C6	0.0741 (15)	0.0634 (13)	0.0630 (14)	−0.0314 (12)	0.0101 (11)	−0.0041 (11)
C7	0.0840 (16)	0.0602 (13)	0.0512 (12)	−0.0235 (12)	−0.0033 (11)	0.0048 (10)
C8	0.0609 (13)	0.0639 (13)	0.0540 (12)	−0.0198 (10)	−0.0100 (10)	0.0006 (10)
C9	0.0513 (11)	0.0483 (10)	0.0474 (10)	−0.0173 (9)	−0.0040 (8)	−0.0042 (8)
Cl1	0.1675 (9)	0.0837 (5)	0.0523 (4)	−0.0326 (5)	0.0147 (4)	−0.0069 (3)
N1	0.0494 (9)	0.0588 (10)	0.0518 (10)	−0.0203 (8)	−0.0065 (7)	0.0006 (8)
N2	0.0465 (9)	0.0509 (9)	0.0520 (9)	−0.0170 (7)	−0.0082 (7)	−0.0028 (7)

### Geometric parameters (Å, °)

**Table:**

C1—C2	1.488 (3)	C4—N2	1.377 (3)
C1—Cl1	1.786 (2)	C4—C5	1.389 (3)
C1—H1A	0.9700	C4—C9	1.398 (3)
C1—H1B	0.9700	C5—C6	1.373 (3)
C2—N1	1.310 (3)	C5—H5	0.9300
C2—N2	1.363 (3)	C6—C7	1.397 (4)
C3—N2	1.453 (3)	C6—H6	0.9300
C3—H3A	0.9600	C7—C8	1.373 (3)
C3—H3B	0.9600	C7—H7	0.9300
C3—H3C	0.9600	C8—C9	1.390 (3)
C3—H3D	0.9600	C8—H8	0.9300
C3—H3E	0.9600	C9—N1	1.393 (3)
C3—H3F	0.9600		
			
C2—C1—Cl1	110.86 (15)	H3B—C3—H3F	56.3
C2—C1—H1A	109.5	H3C—C3—H3F	141.1
Cl1—C1—H1A	109.5	H3D—C3—H3F	109.5
C2—C1—H1B	109.5	H3E—C3—H3F	109.5
Cl1—C1—H1B	109.5	N2—C4—C5	131.59 (19)
H1A—C1—H1B	108.1	N2—C4—C9	105.69 (17)
N1—C2—N2	114.14 (18)	C5—C4—C9	122.71 (19)
N1—C2—C1	123.36 (19)	C6—C5—C4	116.3 (2)
N2—C2—C1	122.49 (18)	C6—C5—H5	121.9
N2—C3—H3A	109.5	C4—C5—H5	121.9
N2—C3—H3B	109.5	C5—C6—C7	121.9 (2)
H3A—C3—H3B	109.5	C5—C6—H6	119.1
N2—C3—H3C	109.5	C7—C6—H6	119.1
H3A—C3—H3C	109.5	C8—C7—C6	121.5 (2)
H3B—C3—H3C	109.5	C8—C7—H7	119.3
N2—C3—H3D	109.5	C6—C7—H7	119.3
H3A—C3—H3D	141.1	C7—C8—C9	117.9 (2)
H3B—C3—H3D	56.3	C7—C8—H8	121.1
H3C—C3—H3D	56.3	C9—C8—H8	121.1
N2—C3—H3E	109.5	C8—C9—N1	130.36 (19)
H3A—C3—H3E	56.3	C8—C9—C4	119.77 (19)
H3B—C3—H3E	141.1	N1—C9—C4	109.87 (17)
H3C—C3—H3E	56.3	C2—N1—C9	104.17 (16)
H3D—C3—H3E	109.5	C2—N2—C4	106.13 (16)
N2—C3—H3F	109.5	C2—N2—C3	128.34 (18)
H3A—C3—H3F	56.3	C4—N2—C3	125.52 (17)
